# Increased all-cause mortality following distal radius fractures in Danish adults: a register-based matched cohort study

**DOI:** 10.2340/17453674.2026.45786

**Published:** 2026-05-09

**Authors:** Cecilie R BUDTZ, Antti P LAUNONEN, Bakir O SUMREIN, Stig BRORSON, Helle K ØSTERGAARD, Merete N MADSEN

**Affiliations:** 1 DEFACTUM, Central Denmark Region, Denmark; 2 Department of Sensory, Neural, and Musculoskeletal Medicine, Musculoskeletal Diseases Centre, Tampere University Hospital, Tampere; 3 Faculty of Medicine and Health Technology, Tampere University, Tampere, Finland; 4 Centre for Evidence‑Based Orthopaedics, Zealand University Hospital, Køge; 5 Department of Clinical Medicine, University of Copenhagen, Copenhagen; 6 Department of Orthopaedics, Viborg Regional Hospital, Viborg; 7 University Clinic for Interdisciplinary Orthopaedic Pathways (UCOP), Elective Surgery Centre, Silkeborg Regional Hospital, Silkeborg, Denmark

## Abstract

**Background and purpose:**

Distal radius fractures (DRFs) are common in older adults and are associated with osteoporosis and underlying frailty. While hip and spine fractures are associated with increased mortality, the association between DRFs and all-cause mortality remains less clear. We aimed to assess all-cause mortality risk following DRF in Denmark compared with age- and sex-matched controls.

**Methods:**

We conducted a nationwide, register-based matched cohort study using data from the Danish National Patient Register and Civil Registration System (1998–2018). Adults (≥ 18 years) with a DRF were matched 1:5 to controls by sex and birthdate ± 30 days. Mortality rates per 10^3^ persons and mortality rate ratios with 95% confidence intervals (CIs) were calculated at 30, 90, 365, and 730 days after the fracture.

**Results:**

We included 190,513 patients with DRF and 952,565 controls (70% female, mean age 59 years). Mortality rates were higher for patients with DRF compared with controls across all age categories, particularly in men and within the first 30 days. Among men, mortality rates ranged from 0.36 to 52.9 per 1,000 persons in patients with DRF and from 0.07 to 20.0 per 1,000 persons in controls. Among women, mortality ranged from 0.16 to 26.3 per 1,000 persons in patients with DRF and from 0.04 to 15.9 per 1,000 persons in controls. Mortality rate ratios were highest for the 50–59 age category (male: 4.60, CI 3.95–5.35, female 3.08, CI 2.80–3.39), decreasing with higher age. Mortality rate ratios stabilized after 365 and 730 days.

**Conclusion:**

This nationwide study shows increased all-cause mortality among patients with DRF compared with sex and age-matched controls, with the highest mortality rate within the first 30 days after the fracture. The mortality rate ratio was consistently higher in the age-group 50–59 and among males.

Distal radius fractures (DRFs) are among the most common adult fractures, and the incidence is rising [1-3]. DRFs account for approximately 17% of all fractures and are especially prevalent in postmenopausal women due to age-related bone loss and increased fall risk [1,2].

While sustaining a hip fracture is clearly associated with an excess mortality [4,5], the association between DRFs and all-cause mortality remains less clear. Unlike hip fractures, which are associated with immobility and complications such as thromboembolism or pneumonia, DRFs are considered less severe. However, studies report an increased probability of frailty in patients with osteoporotic fractures [6,7], and DRFs may signal underlying vulnerability and predisposition to future fractures, potentially influencing mortality [3,8,9].

Previous studies on mortality after distal radius fractures (DRFs) show inconsistent findings. A Danish nationwide study reported increased mortality compared with age- and sex-matched controls [10], whereas other studies have found no difference or even lower mortality in certain subgroups [8,11]. Sex-specific differences have also been reported, with some studies showing higher mortality in women and others in men [12,13].

The Danish national registries provide comprehensive data on DRF diagnoses and mortality, offering a unique opportunity to examine this association in a large, well-defined population. Therefore, the aim of our study was to determine whether adults with DRFs in Denmark have higher all-cause mortality than age- and sex-matched controls.

## Methods

### Study design

This was a register-based matched cohort study. The study was reported according to the RECORD statement [14].

### Data source

Denmark has a population of approximately 6 million people, all of whom have access to universal single-payer healthcare, including free emergency services, inpatient care, and outpatient visits. Nearly all patients with DRF are diagnosed and treated at public hospitals. Although a minority of patients may receive treatment in private hospitals, all fractures confirmed through radiographic evaluation, regardless of the setting, must be registered in the Danish National Patient Register (DNPR) [15]. In this study, we retrieved data from the DNPR and the Danish Civil Registration System (DCRS) [15,16]. The DNPR provides information such as diagnoses, surgical procedures, treatments, complications, and hospital admissions [15]. The validity of DRF diagnoses in the DNPR is high, with a reported positive predictive value of 0.99 [17]. After identifying the DRF population in the DNPR, it was linked to the DCRS using a personal identification number [16]. The DCRS assigns every citizen a unique 10-digit personal identification number at the time of birth, which allows for unambiguous individual-level record linkage to the DNPR and other registries in Denmark. The DCRS provides information on date of birth, sex, and vital status (dead or alive), and was used to identify age and sex-matched controls from the general population.

### Population

The fracture group consisted of all adults (≥ 18 years) with a registered diagnosis of DRF or DRF combined with distal ulna fracture (International Classification of Diseases, 10th Revision: S.52.5* or S.52.6*) in DNPR during a 21-year timespan (1998–2018). Only the patients’ first fracture in the study period was included. The fracture group was matched 1:5 to the background population. The matching criteria were sex and date of birth ± 30 days. If 5 matches could not be found, fracture cases were excluded. Each patient with a DRF was assigned an index date corresponding to the fracture date, and the matched controls were assigned the equivalent index date.

Patients and controls could appear only once in the dataset to ensure data independence: each individual contributed either as a DRF patient or as a population control, but not both. To minimize survival bias, we aligned the data prospectively and performed the matching in chronological order, with the earliest index dates matched first. For each incident DRF, controls were sampled from the general Danish population who were alive, resident in Denmark, and without a registered DRF at the index date of the fracture. Controls were selected based only on information available up to the index date and were not required to remain fracture-free during follow-up; individuals who later sustained a first DRF therefore remained in the control group and were not re-entered as DRF patients.

### Variables

The outcome was exact date of death registered in DCRS within a timeframe of 730 days from the index date. The outcomes included all-cause death episodes. Covariates were age group at index date (18–39, 40–49, 50–59, 60–69, 70–79, 80–89, and > 90) and sex. Additionally, for descriptive purposes, data on whether surgery was performed within 30 days after the index date was collected (only fracture population).

### Statistics

Descriptive demographic characteristics were presented as mean with standard deviation (SD) or proportions. Overall cumulated mortality in the DRF and control groups was presented as number (%) in the restricted time periods 0–30, 31–90, 91–365, and 366–730 days.

Poisson regression analysis accounting for interactions between the group (DRF or control) and restricted follow-up periods (0–30, 31–90, 91–365, and 366–730 days) was performed. Age group and sex-specific mortality rates per 1,000 persons with 95% confidence intervals (CI) were calculated for both DRFs and controls. Mortality rate ratios with CI were calculated for the age groups 50–59, 60–69, 70–79, 80–89, and ≥ 90. MRRs were only calculated from 50 years because this is the internationally recognized lower limit for screening patients with low-energy fractures through the Fracture Liaison Service [18].

To analyze potential time trends in mortality over the 21 years, stratified analyses were performed presenting mortality rate ratios with CI divided into year of the fracture: 1998–2002, 2003–2008, 2009–2013, and 2014–2018 for the age groups 50–59, 60–69, 70–79, and ≥ 80. Model fit was evaluated by comparing the Pearson χ² statistic with its residual degrees of freedom. As this indicated overdispersion, Poisson models were estimated with robust (sandwich) standard errors when calculating CIs. This did not alter the estimated mortality rate ratios and their CIs when rounded to 2 decimals.

Data cleaning, preprocessing, and all statistical analyses were performed using the Stata software package (version 17, StataCorp LLC, College Station, TX, USA). Figures were created in Microsoft Excel 365 (Microsoft Corp, Redmond, WA, USA).

### Ethics, data sharing plan, funding, use of AI, and disclosures

Data access was gained through the Danish Health Data Authority, where Danish health data is collected, stored, and managed. Approval was gained from the Danish Data Protection Agency (reference no. 680317, case no. 1-16-02-100-20). No funding was obtained for this study. ChatGPT (OpenAI) was used for language editing and wording support in the manuscript and response to reviewers. All scientific content, statistical analyses, interpretations, and conclusions were made by the authors, who take full responsibility for the final manuscript. The authors have no conflict of interest to declare. Complete disclosure of interest forms according to ICMJE are available on the article page, doi: 10.2340/17453674.2026.45786

## Results

**Figure 1:**
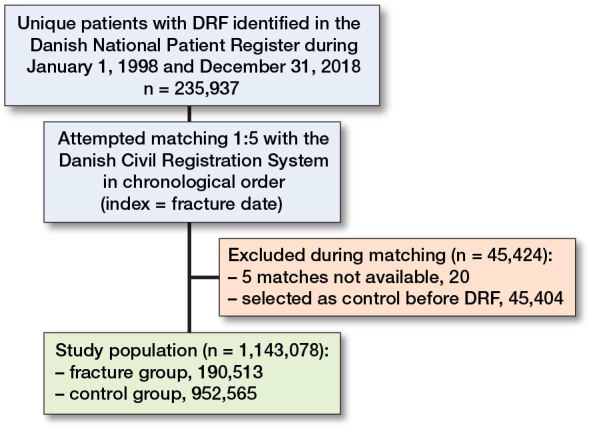
Data management flowchart of study population. DRF: distal radius fracture.

235,937 unique patients with DRF were identified in the study period. After matching, the total study population consisted of 190,513 patients with DRF and 952,565 controls (Figure 1). 20 cases could not be matched with 5 controls and were excluded, and a further 45,404 potential cases were excluded due to being randomly selected as controls before their fracture. Among the 45,000 excluded individuals, all of whom later sustained a DRF, 9,355 (21%) had their DRF, and 554 died (1%) within 2 years after their matching date as a control. The mean age of those excluded was 67 (SD 14).

70% of the population was female, and the population mean age was 59 (SD 19). Participant demographics are displayed in Table 1.

**Table 1:** Characteristics of the study population at index date. Values are count (%)

	DRF	Control
	(n = 190,513)	(n = 952,565)
Female	133,722 (70)	668,610 (70)
Male	56,791 (30)	283,955 (30)
Age groups		
18–39	32,559 (17)	162,792 (17)
40–49	20,251 (11)	101,277 (11)
50–59	37,019 (20)	185,341 (20)
60–69	40,870 (21)	204,265 (21)
70–79	33,825 (18)	168,940 (18)
80–89	21,376 (11)	104,936 (11)
≥ 90	4,613 (2.4)	23,014 (2.4)
Treatment		
Non-surgical	150,723 (79)	–
Surgical	39,790 (21)	–

DRF = distal radius fracture.

The overall proportion of deaths in the DRF groups was notably higher than in the control groups in all the restricted time periods (Table 2). This was particularly pronounced among male DRF cases compared with controls.

**Table 2:** Number of deaths in restricted time periods stratified by sex and divided into age categories. Values are count (%)

Time period	Total	Male controls	Male DRF	Female controls	Female DRF
	(n = 1,143,078)	(n = 283,955)	(n = 56,791)	(n = 668,610)	(n = 133,722)
0–30 days	3,154 (0.3)	479 (0.2)	324 (0.6)	1,617 (0.2)	734 (0.6)
Age groups					
18–39	20 (0.63)	< 10	12	< 10	< 10
40–49	30 (0.95)	11	10	< 10	< 10
50–59	142 (4.50)	43	30	55	14
60–69	298 (9.45)	60	28	141	69
70–79	747 (23.7)	128	82	383	154
80–89	1,217 (38.6)	161	116	627	313
≥ 90	700 (22.2)	73	46	403	178
31–90 days	5,269 (0.5)	926 (0.3)	344 (0.6)	3,117 (0.5)	882 (0.7)
91–365 days	22,361 (2.0)	4,016 (1.4)	1,263 (2.2)	13,952 (2.1)	3,130 (2.3)
366–730 days	29,164 (2.6)	5,362 (1.9)	1,549 (2.7)	18,195 (2.7)	4,058 (3.0)

DRF = distal radius fracture.

**Figure 2:**
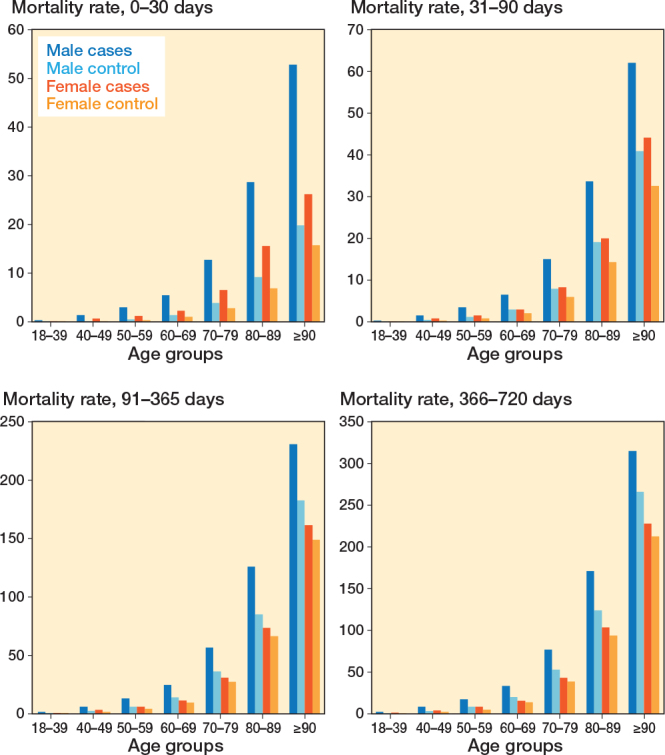
Expected mortality rate per 1,000 persons stratified on restricted time periods and sex.

Mortality rates increased with age for both sexes, with males showing higher mortality rates than females (Figure 2 and Table 3). Patients with DRF had consistently higher mortality rates than controls across all the restricted time periods.

**Table 3:** Mortality rates with 95% confidence intervals for cases and controls stratified on restricted time periods and sex (per 1,000 persons)

Follow-up/age	Male control	Male DRF	Female control	Female DRF
0–30-day				
18–39	0.07 (0.06–0.07)	0.36 (0.31–0.41)	0.04 (0.03–0.04)	0.16 (0.14–0.19)
40–49	0.25 (0.25–0.30)	1.44 (1.27–1.64)	0.16 (0.15–0.17)	0.70 (0.63–0.78)
50–59	0.65 (0.59–0.71)	2.97 (2.64–3.35)	0.41 (0.38–0.43)	1.25 (1.15–1.35)
60–69	1.48 (1.35–1.63)	5.56 (4.94–6.24)	1.01 (0.96–1.07)	2.40 (2.22–2.59)
70–79	3.95 (3.60–4.33)	12.9 (11.5–14.5)	2.91 (2.77–3.06)	6.62 (6.15–7.14)
80–89	9.31 (8.48–10.2)	28.8 (25.6–32.3)	7.03 (6.68–7.38)	15.7 (14.6–16.9)
≥ 90	20.0 (18.2–22.0)	52.9 (46.3–60.5)	15.9 (15.1–16.7)	26.3 (32.0–37.4)
31–90-day				
18–39	0.13 (0.12–0.15)	0.42 (0.37–0.48)	0.07 (0.07–0.08)	0.21 (0.18–0.24)
40–49	0.56 (0.52–0.60)	1.69 (1.50–1.91)	0.33 (0.31–0.35)	0.90 (0.81–0.99)
50–59	1.33 (1.24–1.42)	3.50 (3.13–3.91)	0.83 (0.80–0.87)	1.59 (1.48–1.72)
60–69	3.05 (2.85–3.26)	6.54 (5.85–7.30)	2.08 (2.01–2.16)	3.06 (2.86–3.28)
70–79	8.11 (7.59–8.66)	15.1 (13.6–16.9)	5.99 (5.78–6.21)	8.46 (7.91–9.06)
80–89	19.1 (17.9–20.4)	33.8 (30.3–37.7)	14.5 (14.0–15.0)	20.1 (18.8–21.5)
≥ 90	41.1 (38.2–44.2)	56.5 (54.7–70.8)	32.6 (31.4–33.9)	44.2 (41.2–47.5)
91–365-day				
18–39	0.60 (0.56–0.64)	1.56 (1.40–1.73)	0.34 (0.31–0.36)	0.76 (0.67–0.86)
40–49	2.48 (2.36–2.60)	6.29 (5.79–6.83)	1.52 (1.44–1.58)	3.26 (3.02–3.53)
50–59	5.91 (5.68–6.15)	13.0 (12.1–13.9)	3.80 (3.71–3.89)	5.80 (5.52–6.09)
60–69	13.6 (13.1–14.1)	24.3 (22.7–25.9)	9.51 (9.32–9.71)	11.2 (10.7–11.6)
70–79	36.1 (34.9–37.3)	56.2 (52.8–59.9)	27.4 (26.9–28)	30.8 (29.7–32.0)
80–89	85.0 (82.2–88.0)	126 (118–134)	66.0 (64.8–67)	73.1 (71.4–75.9)
≥ 90	182 (174–191)	191 (210–254)	149 (146–152)	161 (154–169)
366–730-day				
18–39	0.87 (0.82–0.93)	2.12 (1.92–2.34)	0.48 (0.44–0.52)	1.07 (0.94–1.22)
40–49	3.61 (3.44–3.78)	8.57 (7.92–9.28)	2.16 (2.06–2.26)	4.62 (4.28–4.99)
50–59	8.62 (7.81–8.95)	17.7 (16.6–18.9)	5.42 (5.30–5.55)	8.22 (7.85–8.61)
60–69	19.8 (19.1–20.4)	33.1 (31.1–35.2)	13.6 (13.3–13.8)	15.8 (15.2–16.4)
70–79	52.6 (51.0–54.3)	76.7 (72.3–81.3)	39.0 (38.4–39.7)	43.7 (42.2–45.2)
80–89	124 (120–128)	171 (161–182)	94.1 (94.7–95.7)	104 (100–107)
≥ 90	266 (255–279)	315 (287–3456)	212 (208–217)	228 (219–238)

DRF = distal radius fracture.

**Figure 3:**
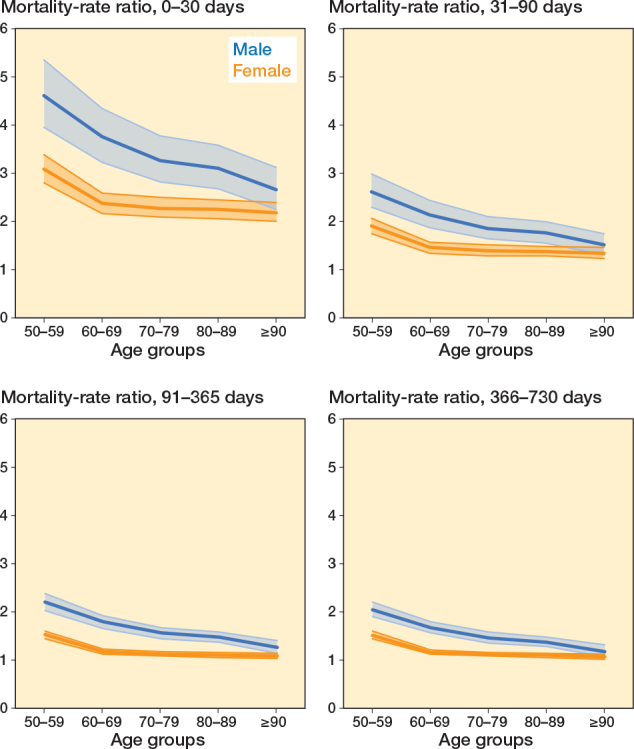
Mortality rate ratio stratified on restricted time periods and sex. Shaded area shows 95% confidence interval.

The mortality rate ratios between patients with DRF and controls were highest within the first 30 days, particularly for the lower age groups, where the age group 50–59 had mortality rate ratios of 4.60 (CI 3.95–5.35) for males and 3.08 (CI 2.80–3.39) for females. The 30-day mortality rate ratios consistently decreased with advancing age groups, resulting in a mortality rate ratio of 2.65 (CI 2.24–3.12) for males and 2.18 (CI 1.99–2.39) for females in the ≥ 90 group (Figure 3 and Table 4).

**Table 4:** Mortality rate ratio with 95% confidence interval stratified on restricted time periods and sex

Follow-up/Age	Males	Females
0–30-day		
50–59	4.60 (3.95–5.35)	3.08 (2.80–3.39)
60–69	3.75 (3.23–4.35)	2.37 (2.16–2.59)
70–79	3.26 (2.82–3.78)	2.27 (2.08–2.49)
80–89	3.09 (2.67–3.58)	2.24 (2.05–2.45)
≥ 90	2.65 (2.24–3.12)	2.18 (1.99–2.39)
31–90-day		
50–59	2.63 (2.31–3.00)	1.91 (1.76–2.08)
60–69	2.14 (1.89–2.44)	1.47 (1.36–1.59)
70–79	1.87 (1.65–2.12)	1.41 (1.30–1.52)
80–89	1.77 (1.56–2.01)	1.39 (1.29–1.50)
≥ 90	1.52 (1.31–1.76)	1.35 (1.25–1.47)
91–365-day		
50–59	2.20 (2.03–2.38)	1.53 (1.44–1.61)
60–69	1.79 (1.66–1.93)	1.17 (1.12–1.23)
70–79	1.56 (1.45–1.68)	1.13 (1.08–1.18)
80–89	1.48 (1.37–1.59)	1.11 (1.06–1.16)
≥ 90	1.26 (1.14–1.41)	1.08 (1.03–1.14)
365–730-day		
50–59	2.05 (1.90–2.21)	1.52 (1.44–1.60)
60–69	1.67 (1.56–1.79)	1.16 (1.12–1.22)
70–79	1.46 (1.36–1.58)	1.12 (1.08–1.16)
80–89	1.38 (1.29–1.48)	1.10 (1.06–1.14)
≥ 90	1.18 (1.07–1.31)	1.07 (1.02–1.13)

Mortality rate ratios were persistently higher among males than females. For both sexes, the mortality rate ratios consistently decreased during the later restricted time periods for all age groups with mortality remaining approximately 2-fold higher among males and 1.5-fold higher among females at 2 years in the 50–59-year age group.

The time-trend analysis revealed a tendency towards higher mortality rate ratios, especially in the older age groups over the 21-year study period (Table 5).

**Table 5:** Time trends in mortality rate ratio with 95% confidence interval

Year	Age 50–59	Age 60–69	Age 70–79	Age ≥ 80
1998–2002	3.89 (3.26–4.64)	2.46 (2.11–2.83)	2.31 (2.01–2.64)	2.11 (1.86–2.40)
2003–2008	4.45 (3.65–5.42)	3.34 (2.81–3.98)	2.65 (2.26–3.11)	2.45 (2.11–2.85)
2009–2013	4.58 (3.68–5.70)	2.93 (2.42–3.56)	2.67 (2.22–3.20)	2.32 (1.96–2.76)
2014–2018	4.12 (3.30–5.15)	3.11 (2.55–3.79)	2.84 (2.35–3.43)	2.59 (2.16–3.10)

## Discussion

This is the largest study to date investigating mortality following DRF, based on a large, precisely matched cohort. We provide new evidence that DRFs are associated with both short- and long-term excess mortality. We found that patients with DRFs had higher all-cause mortality than age- and sex-matched controls, particularly within the first 30 days after fracture. This increased mortality risk was more pronounced in men and in the age group 50–59 years.

A direct comparison of our study with others is difficult due to divergence in populations and methods. However, most comparable with regards to the study population, and matching and analyzed time periods is the study by Christensen et al., who found an increased 30-day mortality risk following DRFs with hazard ratios (HR) of 2.24 (CI 1.94–2.59) for females and 2.52 (CI 1.90–3.34) for males in a Danish context [10]. These results are very similar to ours, although we generally find slightly higher mortality rate ratios and have stratified the analyses into age categories. The tendency is, however, the same as in our study, as their results show consistently decreasing hazard ratios at 90 days, 365 days, and 730 days’ mortality. In our study, we provide age- and sex-stratified mortality estimates, including outcomes for a large cohort of patients aged under 60 years, thereby providing new data on a younger DRF population not previously reported by Christensen et al. We believe this offers important knowledge, as excess mortality after DRF appears to be particularly high in this age group. In addition, we matched cases and controls on date of birth ± 30 days, aiming for greater precision than matching by birth year. Studies on other similar populations from Scandinavia have not found the same increase in mortality after DRF. In contrast, Øyen et al. reported no increased in mortality at 1-year follow-up compared with the background population in Norway but found a significant higher 5-year mortality in female DRF patients over 70 years with a standardized mortality ratio of 1.9 (CI 1.1–2.6) [12]. The authors did not find an increase in mortality at 1 year; however, their study included a relatively small study population (N = 883), which may have limited its statistical power. As a contrast to our results, Arvidsson et al. found lower 1-year mortality in DRF patients aged over 80 years compared with matched controls from the Swedish background population [8]. This study included only 240 fracture cases from a specific area in Sweden, and a possible explanation for the contrast between results could be differences in matching, where they used an indirect standardization in 5-year age groups to the standard mortality rate of the Swedish population.

### Strengths

First, the study is based on a large, nationwide cohort comprising data from the entire Danish background population, minimizing selection bias and ensuring high generalizability. We included 190,513 patients with DRF and 952,565 matched controls. The substantial sample size enables precise estimations of mortality risk and allows for robust subgroup analyses across different age groups and time periods. Furthermore, the use of comprehensive, high-quality register data ensures a high level of data validity. The registers provide complete nationwide follow-up on diagnoses and mortality, reducing the risk of misclassification and loss to follow-up.

A key methodological advantage is the rigorous matching strategy, where patients with DRF were matched 1:5 to the general population based on sex and date of birth ± 30 days. This approach ensures that comparisons were made within individuals of the same age and sex, which are among the most important determinants of mortality. Additionally, survival bias was minimized by aligning the data prospectively before matching, ensuring that controls who later sustained a DRF remained in the control group. This approach prevents potential bias from individuals who had a higher likelihood of surviving long enough to experience a DRF. The inclusion of a time-trend analysis further strengthens the study by providing insights into changes in DRF-related mortality patterns over a 21-year period (1998–2018).

### Limitations

A major limitation is the lack of information on comorbidities and overall health status in both the DRF and control populations. As chronic conditions such as cardiovascular disease, diabetes, and frailty strongly influence mortality [20,21], the inability to adjust for comorbidities introduces a potential source of residual confounding. It is likely that patients with DRF had a higher burden of underlying health conditions compared with controls. Therefore, the observed increased mortality risk might reflect these comorbidities rather than the fracture itself. Even though we do not make any causal assumptions in the results, a more detailed matching strategy, incorporating comorbidity data, could have enhanced the study’s ability to isolate the effect of DRF on mortality. In addition, our DRF population included patients with concurrent fractures, which we chose not to exclude because comparable information on concomitant injuries was not available for matched controls. Excluding these patients only from the fracture population, without equivalent data for the controls, could have introduced selection bias and affected the comparability between groups, while including them allows the study to better reflect the full spectrum of patients presenting with DRF in a broader population. We did not perform analyses stratified by treatment subtypes, as observed differences in mortality between these subgroups would likely reflect variations in patient selection, clinical decision-making, and treatment preferences rather than the intrinsic mortality risk associated with each treatment type. Furthermore, because each individual could contribute to the dataset only once, approximately 19% (45,000/235,937) of potential DRF cases were not included in the fracture cohort, as they had previously been sampled as population controls. These excluded individuals were somewhat older on average (mean age 67 years) than the study population as a whole and may to some extent represent DRFs occurring later in life. Consequently, our fracture cohort is slightly enriched for earlier-occurring DRFs, whereas the control cohort includes some individuals who subsequently sustain a first DRF. To the extent that a propensity to sustain a DRF reflects underlying comorbidity or frailty, this is expected to dilute, rather than exaggerate, the mortality difference between patients with DRF and controls, and our estimates of excess mortality are therefore likely to be conservative. However, this study still provides an important population-level estimate of increased mortality risk in the group of patients with DRF, which is relevant for clinicians and healthcare policymakers. Also, accounting for other potential confounders such as socioeconomic status, medication use, lifestyle factors, or functional status could have further improved the comparability between patients with DRF and controls. For instance, it can be speculated as to whether mental health and lifestyle factors such as alcohol consumption, smoking, and physical activity level might contribute to the high excess mortality observed among the middle-aged, but we cannot determine if they actually have an impact on our results without the relevant data.

### Conclusion

Our study provides evidence of increased mortality following DRFs in a nationwide population-based cohort. Patients with DRF demonstrated higher all-cause mortality rates than their age- and sex-matched controls, with the highest mortality rate within the first 30 days after the fracture. The mortality rate ratios were consistently higher in the age group 50–59 and among males.

*In perspective,* these findings likely reflect underlying patient characteristics and comorbidity rather than a direct effect of the fracture itself, suggesting that a DRF may serve as a marker of increased vulnerability and health risk. From this perspective, a DRF may represent an opportunity for systematic clinical assessment. Post-fracture evaluation through a Fracture Liaison Service, including osteoporosis assessment, could help identify high-risk individuals and guide interventions aimed at reducing subsequent fracture risk and improving overall outcomes.

## Notes

All authors planned and designed the study. CRB undertook data management, conducted statistical analyses, and drafted the manuscript. All authors contributed to interpretation of the results and critical revision of the manuscript. All authors read and approved the final manuscript.

Handling co-editors: Cecilia Rogmark and Philippe Wagner

*Acta* thanks Isam Atroshi and Ida Neergård Sletten for help with peer review of this manuscript.
